# Understanding the Experiences of Black Women Medical Students and Residents: A Narrative Review

**DOI:** 10.3389/fpubh.2022.879135

**Published:** 2022-06-14

**Authors:** Sacha Sharp, Ashley Hixson, Julia Stumpff, Francesca Williamson

**Affiliations:** ^1^Department of Medicine, Indiana University School of Medicine, Indianapolis, IN, United States; ^2^College of Education, University of Maryland, College Park, MD, United States; ^3^Ruth Lilly Medical Library, Indiana University School of Medicine, Indianapolis, IN, United States; ^4^Department of Pediatrics, Indiana University School of Medicine, Indianapolis, IN, United States

**Keywords:** Black women, intersectionality, medical education, medical students, residents

## Abstract

**Background:**

Few research studies examine medical students and residents with intersectional identities. In the emerging literature, data on Black women's experiences may be misrepresented and misinterpreted as studies aggregate data for women, students of color, and Black/African American men. As such, these studies do not account for the nuanced experiences of gendered racism that Black women students and residents may encounter during their medical education.

**Methods:**

Using Crenshaw's intersectionality as an analytical tool, we conducted a narrative review to highlight how Black women medical students and residents are rendered invisible in the current literature on medical education.

**Results:**

The results generated 13 citations specifically discussing Black women medical students and residents, with only six studies being empirical research.

**Conclusion:**

We conclude that 13 articles is inadequate for understanding the experiences of these populations. Without centering Black women or using an intersectional lens, researchers could invalidate the lived experiences of this population and create barriers to the political resources Black women learners need to be successful. Moreover, the lack of intention behind addressing the needs of Black women can be viewed as complicity in the oppressive structures that serve to subjugate them.

## Introduction

Significant research calls for diversity in medical education (1–3). It is understood that a diverse physician workforce contributes to health equity (4). Physicians of color also contribute to the instruction of culturally engaging pedagogies that support medical student success (4, 5). Yet, medical schools have failed to increase diversity even when establishing it as a pillar of educational excellence (6). There is also a dearth of research that explores the experiences of racially and ethnically diverse medical students and residents, specifically Black women.

During the 2021–2022 enrollment period, Black women made up 9.4% of women in medical school (7). This is compared to 8.9% the prior year. Moreover, Black women made up 4.6% (2020–2021), and 4.5% (2021–2022) of the total enrollment population, statistics that have remained relatively stagnant over time. Comparatively, Black women only make up 2% of the active physician workforce (8). Numbers inconsistent with the approximately 6.8% of Black women represented in society (9). Herein lies an issue given that patients disproportionately seek out physicians with shared gender and racial backgrounds (10). Furthermore, without a deeper understanding about the experiences of Black women medical students and residents, institutions of academic medicine will continue to have difficulty with recruitment, and more importantly, retention efforts.

## Review of Literature

Medicine research confirms that medical students and residents of color experience discrimination in the form of racism, microaggressions, and lack a sense of belonging (5, 11, 12). Researchers also confirms gender discrimination experienced by women in medicine (13). Yet, there remains a paucity of medical research designed specifically to center the experiences of individuals with multiple marginalized identities. An intersectional lens is necessary for critically examining the experiences of medical students and residents, which can elucidate the experiences of Black women in medical school and residency.

While few articles are useful in providing a general understanding of the landscape of medicine overall for those underrepresented, there are even fewer articles that explicitly capture the nuanced experiences of Black women and how their race and gender intersect. Particularly for Black women, limited research captures the complex experiences they have in relation to their Black men and white women counterparts (14, 15). Moreover, there is insufficient research that centers Black women with the goal of understanding their experiences only, despite their relationship to Black men or White women.

As scholars and researchers engage with and contribute literature on diversity education for minoritized populations, they must consider the intersectional identities of the learners they seek to study. A review of higher education research reveals that Black women hold multiple marginalized identities (such as their race and gender), which negatively impacts how they navigate and experience higher education (16). In addition to microaggressions and feelings of isolation (17–19), Black women also report racist and sexist interactions on campus with faculty, administrators, and their peers (20). Limited research critically examines the ways gendered racism creates narratives that are dismissive of Black women's identities and experiences resulting in scarce representation and visibility of this group in research.

Contrary to nascent higher education literature on Black women's experiences, medical education research on Black women medical students and residents is negligeable. The research that does exist discusses Black women in terms of statistical representations within medical school or residency programs (21, 22). Consistently, the limited literature that exists only captures Black women's experiences as aggregate with minoritized and underrepresented populations. Such literature may include several racial and ethnic groups or white women (23, 24), or highlights the experiences of Black women as patients or physicians (25–27). While we may draw inferences about Black women medical students and residents from such research, we cannot fully understand how Black women conceptualize their medical school journey until we have research that allows them to directly speaks from their lens.

The failure to disaggregate for these populations ignores the intersectional experiences that Black women in medicine face. To explicate more information about the experiences of Black women medical students and residents, we performed a narrative review of literature about the experiences of Black women medical students and residents. Our purpose was to explore whether the experiences of Black women medical students and residents are uniquely different using intersectionality as the method for how we reviewed the existing literature. Our narrative review was guided by the following research questions:

Using Crenshaw's three-dimensional intersectionality framework as a lens, what can be discovered about the experiences of Black women medical students and residents when reviewing published manuscripts that explicitly mention these demographics?

## Positionality

The research team is made up of three women who identify as Black American and one woman who identifies as a non-Hispanic white American. The lineage of the Black women is consistent with members of the African diaspora whose ancestors were brought to America involuntarily for enslavement. The lineage of the non-Hispanic white woman is consistent with members of the European continent whose ancestors voluntarily came to America. The team is made up of three faculty members, two who work in academic medicine and one of whom serves as assistant librarian. The final research member is a doctoral candidate who studies higher education.

The study is significant because the researchers interact with Black women medical students and residents who desire to see themselves and their experiences represented in medical education research. Moreover, the Black women researchers understand the importance of centering the experience of Black women, because they too have encountered challenges associated with navigating academic medicine. As for the white woman, her research areas are library-related, and she has not done research on women or on underrepresented populations in the past. However, as a librarian, she has previously conducted literature searches focused on women and/or underrepresented populations related to medical topics. She identifies as a feminist but does not experience the unique marginalization experienced by women of color.

## Analytical Framework

Following the leads of MacKinnon (28), Haynes et al. (15), and Porter and Byrd (29), we used intersectionality as an analytical tool to highlight details often overlooked in literature about Black women. Intersectionality reflects an interdisciplinary theory designed to capture the complexity associated with social identities and inequities through an integrated approach (30). Intersectionality is said to “refute the compartmentalization and hierarchization of the great axes of social differentiation through categories of gender/sex, class, race, ethnicity, disability, and sexual orientation [(30), p. 58].” In other words, intersectionality recognizes how identities are socially constructed and experienced simultaneously, not hierarchically (14).

Kimberlé Crenshaw (31) introduced the concept of intersectionality by explaining how the lived experiences of Black women were inconsistent within the ideological constraints of mainstream feminist theory. The inconsistency was due in part to the patriarchal expectations white women were trying to overcome, such as working outside the home. Crenshaw exampled how Black women have always been expected to work outside the home, a contradiction to the white woman experiences described by feminist theory (31). Crenshaw also asserted that Black women were not viewed as passive, a generalization made regarding all women by feminist literature, but in fact were often stereotypically viewed as aggressive by mainstream society (31). Therefore, Black women live in a society with sex-based expectations where racism operates simultaneously to deny those expectations.

Crenshaw argued that Black women are often excluded from certain antiracist discourses because those discourses are based on experiences that do not consider how race and gender intersect (14). Crenshaw stated, “with Black women as the starting point, it becomes more apparent how dominant conceptions of discrimination condition us to think about subordination as disadvantage occurring along a single categorical axis [(14), p. 140].” Crenshaw's (14) theory of intersectionality removes the single-axis framework and allows for viewing discrimination and oppression from multiple angles. Thus, intersectionality can be understood using three-dimensions– structural, political, and representational.

The three dimensions of intersectionality are structural, political, and representational. Structural intersectionality calls attention to how Black women are made invisible due to their placement behind Black men racially, or behind white women regarding gender. Black women experience different forms of oppression that go unacknowledged due to Black men and white women being the minoritized default. Political intersectionality is characterized by political agendas failing to recognize the needs of Black women. This form of intersectionality highlights the political assumptions that all Black women have the same allegiances to racial justice or gender justice agendas as their Black men and white women counterparts. Finally, representational intersectionality is defined by how Black women are viewed in the media or discussed in public discourse. It exposes the harmful impact of Black women being relegated to stereotypes and archetypes (e.g., Mammy, Jezebel, and Sapphire; (32)). In the following section, we will discuss the materials used for our narrative review as well as methods. Following this section, our results will outline how the data found through our review is consistent with the three dimensions of intersectionality.

## Materials and Methods

To answer our research question, we conducted a narrative review (33) to identify and synthesize literature on Black women medical students and residents. Although the search for articles in Medline was systematic, this is a narrative review because (a) the search was conducted in only one database (b) study inclusion and coding decisions were not carried out by reviewers who worked independently and then compared answers, (c) studies reviewed were not appraised for methodological quality, and d) the process did not follow all the steps for a systematic review. Additionally, because we were only interested in literature that explicitly discussed Black women medical students and residents, we were not concerned with the possible pitfalls associated with narrative reviews, such as selection bias or articles with conclusions based on opinion (34). We also did not limit the parameters of the search by date as we expected there would be limited medical research that explicitly discussed Black women.

First, we did a preliminary search using a variation of search terms (e.g., “African American woman,” or “Black woman,” and “ medical student,” and “resident,”) through the National Library of Medicine (NLM) principal biomedical bibliographic broad electronic database [MEDLINE (OVID)] in order to determine what works were published and the utility of our search terms. This database was chosen as it houses most of the articles written about medical education in the United States. The preliminary search was conducted on June 3, 2021 and yielded 131 items. Once we determined the appropriate search terms an additional search was performed on July 7, 2021 that yielded 545 additional articles. Therefore, our search revealed 676 articles total. The full search strategy consisted of the following terms:

- “medical school(s)”
- “Medical student(s)”
- “Internship and residency”
- “Learner or trainee”
- “Clinical clerkship or education”
- “Women or woman or female”
- “African American or Black”

### Inclusion Criteria

Manuscripts that included the following criteria were selected for analysis:

1. Explicitly mentioned Black women.
2. Discussed Black women as medical students or residents.
3. Were published in America.
4. Published in English.
5. Available in full-text.

Articles that did not meet this criteria were excluded from the narrative review.

### Study Selection

Material selection consisted of an initial review of articles generated from the electronic database search. We engaged in intercoder reliability, which increased consistency and transparency in our coding process (35). We organized the articles with the code of three for manuscripts that met our inclusion criteria, two for articles deemed potentially relevant, and one for articles that were unrelated therefore did not meet our inclusion criteria.

**Figure 1:**
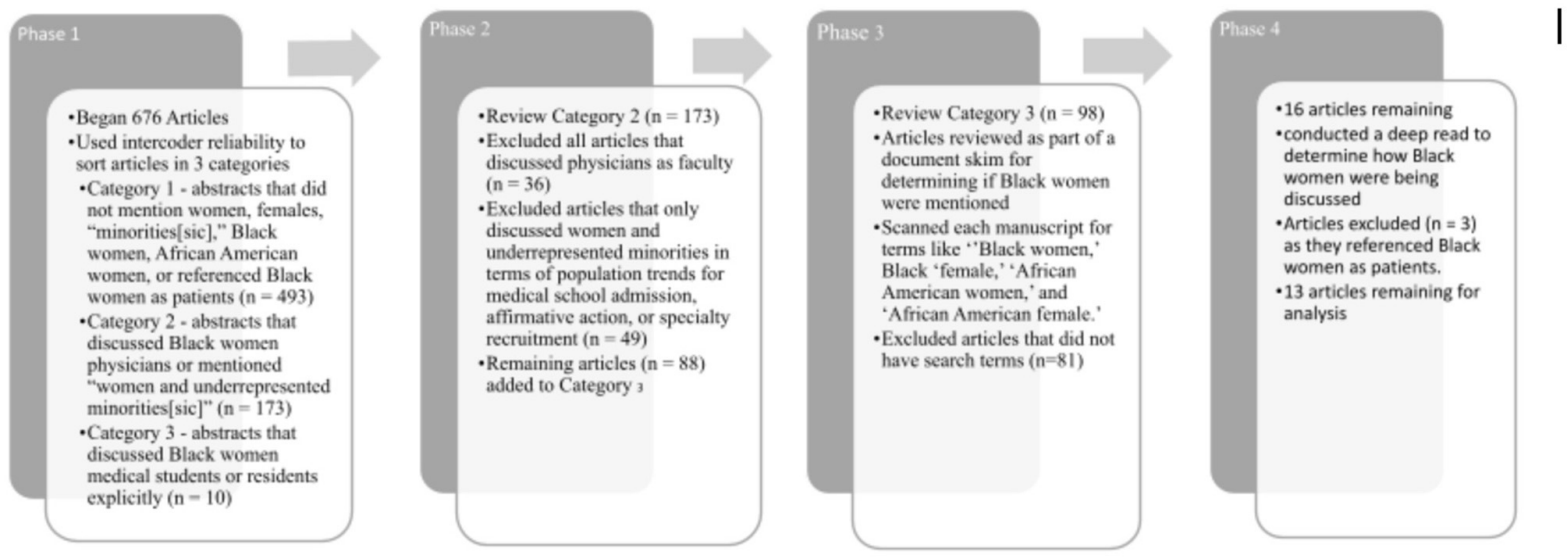
Study selection process.

The selection process included four phases (Figure 1). First, we read the titles and abstracts of each article to determine whether Black women were the focus or even mentioned in the manuscript. Manuscripts with abstracts that did not mention women, females, “minorities[sic],” Black women, African American women, or referenced Black women as patients were assigned a one (1) and excluded from the sample. Manuscripts that discussed Black women physicians or mentioned “women and underrepresented minorities[sic]” were coded as possibly relevant (*n* = 173) and assigned a two (2). Finally, articles that discussed Black women medical students or residents explicitly were assigned a three (3) and considered to meet the inclusion criteria (*n* =10).

For the next phase, we reviewed the 173 abstracts that were considered possibly relevant. We excluded all articles that discussed physicians as faculty rather than residents (*n* = 36) and we excluded articles that only discussed women and underrepresented minorities in terms of population trends for medical school admission, affirmative action, or specialty recruitment (*n* = 49). This exclusion was because these articles did not disaggregate the data to show statistics for Black women specifically, and they were not concerned with the experiences of this population. We added the remaining possible articles (*n* = 88) to the articles that met the inclusion criteria (*n* = 10) creating a total of 98 articles to be reviewed as part of a document skim for determining if Black women were mentioned in the manuscripts.

Two researchers scanned each manuscript for terms like “Black women,” Black “female,” “African American women,” and “African American female.” As a result, we found 16 articles that explicitly mentioned Black women. For the final phase, the two researchers conducted a deep read of the relevant articles to determine how Black women were being discussed, and to make certain the experiences of Black women learners received attention in the manuscript. Upon completion of the deep reads, we found only 13 articles to meet our inclusion criteria. Those articles are referenced below in Table 1. Articles removed as a result of the deep read discussed Black women as patients, nurses, or other support staff. As a research team, we found this process to be sufficient in finding articles about Black women medical students and residents, thus answering our research questions.

**Table 1:** Studies explicitly mentioning Black women medical students and residents.

**#**	**Title**	**Article type**	**Source**	**Author(s)**
1	#Me_Who Anatomy of Scholastic, Leadership, and Social Isolation of Underrepresented Minority Women in Academic Medicine.	Perspective	Circulation. 138(5):451–454, 2018 07 31.	Albert, Michelle A
2	Anticipation of predictable stressors: a course to promote well-being for women physicians of color.	In Progress: Annual Feature	Academic Medicine. 75(5):516, 2000 May.	Goldstone, R Drake, M
3	M. Deborrah Hyde, MD, MS: the second African-American female neurosurgeon.	Guest Editorial	Journal of the National Medical Association. 99(10):1193–5, 2007 Oct.	McClelland, Shearwood 3rd
4	Medical Students Rate Black Female Peers as Less Socially Connected.	Empirical	Journal of the National Medical Association. 110(2):157–162, 2018 Apr.	Royal, Kenneth D
5	Mental Well-Being in First Year Medical Students: A Comparison by Race and Gender: A Report from the Medical Student CHANGE Study.	Empirical	Journal of Racial & Ethnic Health Disparities. 2(3):403–13, 2015 Sep.	Hardeman, Rachel R Przedworski, Julia M Burke, Sara E Burgess, Diana J Phelan, Sean M Dovidio, John F Nelson, Dave Rockwood, Todd van Ryn, Michelle
6	Selected characteristics of black physicians in the United States, 1972.	Empirical	JAMA. 229(13):1758–61, 1974 Sep 23.	Thompson, T
7	They Don't See a Lot of People My Color: A Mixed Methods Study of Racial/Ethnic Stereotype Threat Among Medical Students on Core Clerkships.	Empirical	Academic Medicine. 95(11S Association of American Medical Colleges Learn Serve Lead: Proceedings of the 59th Annual Research in Medical Education Presentations):S58-S66, 2020 11.	Bullock, Justin L Lockspeiser, Tai Del Pino-Jones, Amira Richards, Regina Teherani, Arianne Hauer, Karen E
8	Factors that Influence Underrepresented in Medicine (UIM) Medical Students to Pursue a Career in Academic Pediatrics.	Empirical	Journal of the National Medical Association. 113(1):95–101, 2021 Feb.	Dixon, Gabrina Kind, Terry Wright, Joseph Stewart, Nikki Sims, Alexandra Barber, Aisha
9	Perspectives on Race and Medicine in the NICU.	Perspective	Pediatrics. 147(3), 2021 03.	Adams, Shannon Y Davis, Tanika White Lechner, Beatrice E
10	I Can't Breathe during Interviews - The Incomplete Penetrance of Antiracism.	Perspective	New England Journal of Medicine. 384(19):e72, 2021 05 13.	Kemet, Shakkaura
11	Let's Get Uncomfortable.	Perspective	Annals of Surgery. 273(2):e37–e38, 2021 02 01.	McElroy, Imani
12	Minority Resident Physicians' Views on the Role of Race/Ethnicity in Their Training Experiences in the Workplace.	Empirical	JAMA Network Open. 1(5):e182723, 2018 09 07.	Osseo-Asare, Aba Balasuriya, Lilanthi Huot, Stephen J Keene, Danya Berg, David Nunez-Smith, Marcella Genao, Inginia Latimore, Darin Boatright, Dowin
13	I Am an African American: Distinguishing Between African American and African Applicants in Medical School Admissions Matters.	Perspective	Academic Medicine. 93(9):1281–1285, 2018 09.	Baugh, Reginald F

### Data Analysis

To conduct the analysis, the research team first reread each article to assign codes related to the intersectionality framework. We considered whether the researchers used intersectionality in their analysis, if there were possibilities for considering intersectionality, and how the articles contributed to intersectionality discourses overall. We engaged in a deductive coding process using Crenshaw's three dimensions of intersectionality as our guide. The findings from our analysis are outlined in the results section below.

### Delimitations

The first delimitation is consistent with our search criteria. Because we limited our search to articles published in America that offered full-text, this is not an exhaustive list of literature about Black women medical students and residents. Completing a non-exhaustive search is consistent with aspects of narrative review. Lastly, because of the timeframe for when we conducted our search, we may have missed manuscripts published after July 2021.

## Results

Firstly, it must be noted that there are not many articles that discuss the experiences of Black women medical students and residents. To only have 13 articles is not sufficient for exploring the needs of this demographic and providing meaningful responses to issues that may hamper their success. Nonetheless, we present results of this narrative review to assess the current state of the literature and identify future areas for research. Second, only six of these articles are empirical studies that examine data related to Black women's experiences. Finally, none of these articles use the dimensions of intersectionality to understand how power dynamics impact the lived experiences of Black women in medicine. That said, one article did at least mention intersectionality in terms of how race, gender, and class intersect for Black women who experience stereotype threat in medical school (36).

Of the few articles we found, the results related to the three dimensions of Intersectionality in three ways. The first related to structural intersectionality and concerns the invisibility of Black women in medicine. The second is regarding how Black women remain at the margins of research studies, thus impacting their political needs (14). Finally, the third highlights the harmful implications of how Black women are perceived in society based on stereotypical representations.

### Structural Intersectionality

Structural intersectionality highlights how Black women are rendered invisible due to a focus on Black men and white women (15). Consistent with this dimension, the results of our analysis highlight how the experiences of Black women medical students and residents were marginalized, even when manuscript abstracts suggested Black women would receive more attention. Authors shared how Black people and women were at greater risk of discrimination, stereotype threat, isolation, and poor wellbeing in medical school due to racism and sexism. However, Black men became the default for Black people, and women often referred to white women, leading to Black women's erasure. For example, a study on the wellbeing of first year medical students included a multivariate analysis to determine if individuals at the intersection of gender and race (i.e., Black women) were at higher risks for depression and anxiety (37). The study found that although Black students and women were at greater risk, Black women were not at the same level of risk. The authors went on to suggest Black women were less vulnerable than their Black men counterparts. As for implications, the authors focused more on the results of women and Black students ignoring the possible nuance that made Black women less vulnerable in the data.

In another study, mixed methods were employed to determine the stereotype vulnerability of racially and ethnically diverse medical students (36). The quantitative results highlighted how all participants in the study were at risk of stereotype threat. Qualitatively, interviews with Black women were highlighted. However, the discussion of results did not mention Black women specifically. The authors noted that 80% of the Black respondents experienced stereotype threat and that women commonly faced microaggressions. Yet, little effort was made to discuss the intersectional impacts of these phenomena for Black women. Even when intersectionality was mentioned as a component of critical race theory, the framework used to analyze qualitative results, the authors failed to embrace the knowledge that could be gained when centering Black women.

The articles highlighted represent the structural issues that can be disrupted using an intersectional lens. Moreover, the findings underscore the intersectional subordination Black women often endure. If Black women received more attention in these studies, researchers could further explain how to mitigate risks of stereotype threat, depression, and anxiety for this population. Without understanding the experiences of Black women, researchers could misconstrue results, thus explaining away the need to provide focused attention on Black women's mental health. Doing so would therefore limit Black women medical students' and residents' access to political resources.

### Political Intersectionality

Political intersectionality is the failure to intentionally center Black women in research with the purpose of increasing their access to medical school resources. Several articles defaulted to discussing general diversity and inclusion in the context of those underrepresented in medicine (URM), thus creating competition amongst those assigned the distinction. The lack of intentionality related to increasing the political resources for Black women appeared in two ways, (1) by choosing to focus the needs of URM populations at the onset of study creation when a focus on Black women is apparent, and (2) by ignoring results that highlight the needs of a specific underrepresented group, such as Black women.

An example of lacking intention on the onset of study creation is a study where authors evaluated the social connectedness of students in medical school learning communities (38). The researchers asserted that the study was designed to evaluate the connectedness of peer cohorts across sex and racial groups, which would indicate a focus on Black women. However, it was not until reflection of the results that Black women received attention. Results from the study indicated Black women were less connected and ultimately isolated from their peers. Although the article generated insights into potential reasons why Black women medical students were less socially connected, this would have possibly gone under acknowledged if there were no statistically significant data representations.

Similarly, in an empirical study about the experiences of residents from minoritized populations, qualitative interviews were performed to determine how participants engaged workplace diversity (39). Black women residents shared their experiences with microaggressions and discrimination. One participant discussed being called by other Black women's names as there were six Black women residents in the program. Another Black woman participant shared how their attending suggested Black people have scientifically thicker skin. The women expressed how they felt they could not correct the attending for fear of retribution. Hair was also mentioned by a participant, as they were told their hair made patients uncomfortable. Yet, the ethnicity of this participant was not revealed. These accounts were a part of a larger study with Black, Latinx, and Indigenous Americans. The authors shared implications such as monitoring instances of bias and unburdening minoritized residents with leading diversity initiatives. Because the study was designed to explore the experiences of those underrepresented in medicine, rather than centering the experiences of one group, little attention was paid to the complex lived experiences of each participant, especially Black women. Further, the implications provided left little room for allocating the political resources necessary to enhance the experiences of the specific minoritized groups enduring the most harm.

Finally, in a study designed to explore the experiences of underrepresented medical students interested in pediatrics, results that highlighted the needs of Black women medical students were wholly ignored. Dixon and colleagues conducted three focus groups at Howard University College of Medicine and George Washington University School of Medicine and Health Sciences (40) to learn more about students interested in pediatrics. Themes related to mentorship, familial influences, work, debt, and knowledge about the field were discovered because of the study. However, because 15 of the 18 study participants were Black women, the authors found this to be a limitation to their research. Further, they continued to reference and promote the needs of underrepresented students in medicine rather than focusing on the identities of most of their study participants.

These findings serve as an example of political intersectionality because much of the research assumes the political needs (i.e., resource allocations) of Black women medical students are consistent with their Black men, white women, or URM counterparts. To homogenize Black women within broad categories, such as URM, reinforces systems of oppression and ignores the notion that Black women themselves are not a monolith and do have a diversity of needs. It is not until researchers are intentional about examining the experiences of Black women will their political desires be met.

### Representational Intersectionality

Our last finding is consistent with representational intersectionality. Representational intersectionality underscores the harmful implications from the public's perception of Black women that create monolithic imagery, often stereotypical, of Black women. This finding is associated with articles where Black women report their perception and rationale with how and why they are treated in a particular way, as well as tactics they use to counter the monolithic narratives about their lived experiences. The few articles that emphasized Black women's medical experiences as students and residents highlight the complex challenges with which Black women are confronted that may be attributed to their identities. Prior to discussing these articles, we will elaborate on the various archetypes that have been ascribed to Black women, and the tropes they must fight to overcome.

Collins (32) reminds us that Black women are often relegated to negative archetypes and tropes such as the Mammy, Jezebel, or Sapphire. The Mammy archetype has roots in slavery where the Black woman is depicted as the caregiver who worked for white families. The Jezebel imagery is associated with Black women being seductresses with strong appetites for sex. Finally, the Sapphire archetype represents the boss like Black women whose intentions are to assume the role of men. More current representations of Black women include the Black Lady (41), a role developed to speak to respectability politics and disrupt narratives of Black women's promiscuity. Moreover, there is the Angry Black woman archetype that suggests Black women to be combative and never experiencing joy. For Black women medical students and residents, these stereotypes may have negative implications for them emotionally and psychologically, such as the effects that come with stereotype threat and imposter syndrome.

An example of the harmful impacts of stereotyping that Black women often endure was shared by a resident recounting her OB/GYN residency interviews (42). The resident expressed her need to splinter herself to display “eager optimism, and an unwavering belief” [(42), p. e72] in the transformation of the medical system. This optimism was required even though she held complex emotions of hopelessness regarding the state of medical education. Yet, because she understood the nature of the medical Match system, she avoided displaying emotions that could potentially signal beliefs that she was an “Angry Black woman who couldn't be successfully mentored or taught” [(42), p. e72] as these beliefs could ultimately derail her future career as a physician.

In another perspective piece, McElroy (43) shared how she experienced vitriol on social media for being the only Black surgeon in her residency program. Coupled with events surrounding the murder of George Floyd in 2020, she shared how the experience of representing Black women in the surgery program had overtaken her training. McElroy went on to express how her experiences were mired with negativity because others considered her to be the affirmative action admit, or not deserving of her spot in the surgery program. These types of feelings could translate to imposter syndrome and tokenism. McElroy concluded the piece with suggestions on how to move surgery forward, discussing her treatment in terms of intersectionality and exposing nonracists for their inaction.

These findings offer cogent examples of representational intersectionality. Within each article, Black women reflected on their experiences through an intersectional lens that accounted for both their Blackness and womanhood. The women highlighted the insidious and harmful impact of gendered and racialized stereotypes for Black women. Further, they shared ways medical schools could mitigate barriers to Black women's success giving voice to those who often go underserved.

### Discussion and Recommendations

The purpose of this study was to examine extant literature on Black women medical students and residents to identify gaps and opportunities for research in this area. We asked, what could be discovered about the experiences of Black women learners when reviewing publications that explicitly mention these demographics? Through conducting a narrative review, we discovered there is insufficient research on Black women medical students and residents. Of the empirical studies we found, researchers failed to center the experiences of Black women or ignored specific challenges these women face, thus sustaining the systems that impose structural power over Black women. Our study contributes several insights into the state of the recent literature on this topic:

More research should be devoted to understanding the experiences of Black women medical students and residents.

When researchers fail to center Black women medical students and residents, they cannot fully understand the lived experiences of these populations.

Without an intersectional lens, researchers may miss the nuanced challenges Black women medical students and residents face.

First, this review serves as a call to action for researchers and medical educators. Given the dearth of literature on Black women's experiences in medicine, more research is needed to improve the field's understanding of how to adequately and appropriately provide support that addresses structural, political, and representative barriers to success. To expand this literature base, medical educators and researchers should work with current Black women medical students and residents to understand their experiences. Furthermore, it is essential to underscore the need for empowerment, support, and belonging for Black women medical students and residents. Tuck (44) argued that in efforts to define the problems students from marginalized groups face, education researchers inadvertently perpetuate “damage-centered” narratives that obscure positive aspects of their experiences. Thus, future research should also consider how Black women leverage their strengths to thrive, experience joy, and embody hope across the trajectory of their medical training. Additionally, understanding Black women's experiences in medical education, may lead to identifying effective retention strategies that are relevant and supportive in how they persist.

Second, centering Black women's voices and perspectives is critical to our ability to generate an evidence base that can inform how institutional leaders design and implement initiatives to support their success. For example, we illustrate that the emerging literature base accounts for some of the ways that racism and sexism negatively impact Black women medical student's well-being. Though recent studies offer valuable insights, we argue that by decentering Black women, the analyses risked (mis)interpreting Black women as less vulnerable because they were compared to Black men and White women. As Crenshaw (31) argued, analyses of racism or sexism alone cannot account for how Black women's experience the simultaneity of these oppressions. Thus, the relative comparisons within racial (e.g., Black men), gender (e.g., White women), and URM groups cannot adequately capture Black women's experiences in medicine.

Third, researchers should apply Black feminist and intersectional analytic frameworks [e.g., (32, 45)] that account for the distinctive and varied ways Black women experience racism and sexism, along with other aspects of their identities that may shape their experiences in medicine (e.g., age, appearance, class background, dis/ability, partner status, parenting, sexual orientation). Such studies would require disaggregating data in empirical research and refrain from aggregating that data for all racially and ethnically diverse populations to uncover their nuanced experiences.

## Conclusion

We conducted a narrative review to explore the existing literature that explicitly mentioned the experiences of Black women medical students and residents. The review produced 13 articles that discussed Black women learners, with only six articles being empirical. Using Crenshaw's three dimensions of intersectionality - structural, political, and representational, we performed a deductive analysis on the content represented in each article. We found that the articles did not center the experiences of Black women medical students and residents, nor did they use an intersectional lens when communicating results that involved Black women. We conclude that 13 articles are inadequate for understanding the experiences of these populations. Without centering Black women or using an intersectional lens, researchers could invalidate the lived experiences of this population and create barriers to the political resources Black women learners need to be successful. Moreover, the lack of intention behind addressing the needs of Black women can be viewed as complicity in the oppressive structures that serve to subjugate them.

## Author Contributions

SS, AH, JS, and FW were involved in the concept design, data analysis, review, and revision of this manuscript. SS and AH came up with the initial concept and decided to include JS and FW as they offered relevant expertise. JS took the leading role in data collection and assemblage. SS and AH were involved in crafting the initial manuscript, data analysis, and interpreting the results. SS, AH, and FW were involved in completing the discussion as well as critically reviewing and revising the manuscript for significant intellectual content. All authors approved the submission of the final version of the manuscript.

## Funding

The authors graciously acknowledge the financial support of the Indiana University School of Medicine Office of Diversity Initiatives as this research study was funded as part of the Program to Launch Underrepresented faculty Success (PLUS).

## Conflict of Interest

The authors declare that the research was conducted in the absence of any commercial or financial relationships that could be construed as a potential conflict of interest.

## Publisher's Note

All claims expressed in this article are solely those of the authors and do not necessarily represent those of their affiliated organizations, or those of the publisher, the editors and the reviewers. Any product that may be evaluated in this article, or claim that may be made by its manufacturer, is not guaranteed or endorsed by the publisher.
